# Antimicrobial Stewardship in Tropical Infectious Diseases: Focusing on Dengue and Malaria

**DOI:** 10.3390/tropicalmed7080159

**Published:** 2022-07-30

**Authors:** Ashley Siribhadra, Thundon Ngamprasertchai, Pinyo Rattanaumpawan, Saranath Lawpoolsri, Viravarn Luvira, Punnee Pitisuttithum

**Affiliations:** 1 Department of Clinical Tropical Medicine, Faculty of Tropical Medicine, Mahidol University, Bangkok 10400, Thailand; ashley_siri@hotmail.com (A.S.); viravarn.luv@mahidol.ac.th (V.L.); punnee.pit@mahidol.ac.th (P.P.); 2 Department of Medicine, Faculty of Medicine Siriraj Hospital, Mahidol University, Bangkok 10700, Thailand; pinyo.rat@mahidol.ac.th; 3 Department of Tropical Hygiene, Faculty of Tropical Medicine, Mahidol University, Bangkok 10400, Thailand; saranath.law@mahidol.ac.th

**Keywords:** antimicrobial stewardship, antimicrobial resistance, dengue, malaria, acute undifferentiated febrile illness, rapid diagnostic tests, antibiotics, antimicrobial agents, acute febrile illness

## Abstract

Acute undifferentiated febrile illness (AUFI) is the presenting symptom of various tropical and infectious diseases. Viral infection is generally the most common cause of AUFI, accounting for 8–11.8% of cases; thus, antibiotics might be unnecessary. Dengue and malaria are common tropical infectious diseases requiring effective supportive treatment and antimalarial agents, respectively. The uncertainty of early diagnosis results in widespread empirical antimicrobial treatment in high -income as well as in low-and middle-income countries. Although rapid diagnostic tests (RDTs) have been shown to limit antibiotic prescriptions in dengue and malaria, we observed a wide range of antibiotic prescriptions for 13–92.7% of cases in previous literature, particularly in RDT-negative malaria cases. Given several RDT limitations, antimicrobial stewardship (AMS) appears to be an effective strategy for controlling unnecessary antibiotic use and antimicrobial resistance (AMR) prevention. This program should be endorsed by a multidisciplinary team in tropical diseases to control collateral damage of inappropriate antimicrobial use. Empirical antibiotic treatment should be administered based on clinical judgement, microbiological evidence, and local epidemiological data. Rapid termination of antibiotic therapy, including disease control or elimination, is the mainstay of AMS in tropical diseases. Local and international sectors should implement an AMS programme to reduce AMR in the Tropics.

## 1. Introduction

Acute undifferentiated febrile illness (AUFI) is a common presentation of tropical infections. Dengue is hyperendemic in tropical and subtropical countries [1], predominantly in urban and semi-urban areas [2]. Dengue infection is the most common AUFI etiology in Bangkok, Thailand, accounting for 39.6% of non-malarial febrile cases from 2013 to 2015 [3]. Despite the global malaria recession, it remains the most common AUFI diagnosis in various countries, particularly in Sub-Saharan Africa [4], but was only observed at 1% in Southeast Asia [5]. Distinguishing between tropical infection, such as dengue or malaria, and bacterial infection at the early presentation of AUFI is difficult. The non-specificity of symptoms and signs and lack of availability of diagnostic tests often result in irrational antibiotic use [6,7,8]. Following the overuse of antibiotics, antimicrobial resistance (AMR) has emerged as a serious global public health threat in this century. Increasing AMR rates require urgent attention and intervention by utilizing antimicrobial stewardship (AMS) programs [9]. These programs should be implemented not only for general infectious disease but also for tropical infectious diseases. This review aimed to summarize antibiotic use and the AMS program in tropical infectious diseases, with a focus on dengue and malaria. Furthermore, this study aimed to promote AMS program utilization in tropical infectious diseases.

## 2. Acute Undifferentiated Febrile Illness

### 2.1. Causes

AUFI is a common presentation of tropical infectious diseases or bacterial infections without organ specific infection. This presentation is caused by a diverse range of pathogens and can vary between regions. A review in Southeast and South Asia found that viral etiologies were most common, followed by bacterial. Of the viral causes, dengue fever was the most common, accounting for 11.8% of cases, followed by leptospirosis, typhoid, scrub typhus and influenza [1]. The multinational multicenter cross-sectional study of community-acquired sepsis and severe sepsis among children and adults in Southeast Asia showed that dengue infections, followed by leptospirosis and rickettsiosis, were the most common causative pathogens for sepsis in this area [5]. As dengue infection is caused by a virus, antibiotics are not the key treatment.

In a review study conducted in Sub-Saharan Africa, Marks et al. found that malaria was the most common cause of AUFI in children in Burkina Faso and Sudan [10]. Another study by Kaboré et al. in Burkina Faso found that malaria and acute respiratory infections were the primary causes of AUFI [11]. In Nigeria, a malaria-endemic country, identifying the cause of non-malarial febrile illness is difficult. A prospective study conducted by Popoola et al. in a large city found that malaria accounted for the majority of patients with AUFI, at 27% [12]. This was closely followed by bacteremia, accounting for 17.2% of patients, with *Staphylococcus aureus* followed by *Salmonella* Typhi as the most commonly identified pathogens.

Specific rapid diagnostic tests (RDTs) are required in malaria and dengue, both of which do not need empirical antibiotic therapy. Furthermore, this strategy may directly affect patients and indirectly introduce selective pressure, leading to AMR. The lack of a decision algorithm following negative RDTs in healthcare facilities can increase unnecessary antimicrobial use [13]. The common cause of AUFI and characteristics of RDTs are summarized in Table 1.

### 2.2. Antibiotic Use in AUFI

Monitoring antibiotic use in AUFI is important to improve prescribing practices in the future. Previous literature on the types of antibiotics used in both malaria- and non-malaria- endemic regions was reviewed.

A study conducted in Pune, India explored the use of antibiotics in suspected and diagnosed mosquito-borne illnesses among children and adults with acute febrile illnesses. Robinson et al. demonstrated, that on admission, 82% of adults and 94% of children received empirical antibiotics [19], i.e., the vast majority in both cases. Only 6% of patients were diagnosed with culture-positive bacterial infection, suggesting that a large proportion of antibiotic usage was unnecessary. The high rates of empirical antibiotic prescriptions were attributed to diagnostic uncertainty, even when suspicion of non-bacterial illness prevailed. Third generation cephalosporins were the most frequently prescribed antibiotics, administered to 52% of participants, followed by amoxicillin/clavulanate (28%) and macrolides (20%).

Anand Paramadhas et al. conducted a point prevalence study on antimicrobial use in public and private hospitals in Botswana, where human immunodeficiency virus (HIV) infections, tuberculosis (TB), and malaria were endemic. Cefotaxime followed by parenteral metronidazole were the most commonly prescribed antimicrobial agents in all public hospitals, whereas ceftriaxone was the second highest antimicrobial prescribed in specialist private hospitals [20]. Of 711 patients, approximately 70% had a documented bacterial infection, most commonly pneumonia, skin and soft-tissue infections, intra-abdominal infections, and urinary tract infections. However, malaria was not a potential risk factor influencing antibiotic prescription. Okoth et al. found that antibiotics were frequently used in AUFI, at 17%. The most prescribed antibiotics are third-generation cephalosporins, imidazole derivatives and broad-spectrum penicillin [21].

A point prevalence survey conducted by Guterres et al. in Indonesia identified ceftriaxone (15%), levofloxacin (9.2%) and ampicillin sulbactam (7.9%) as the top three antibiotics administered, mostly for the treatment of community-acquired infections, hospital-acquired infections, and prophylaxis, among inpatients in a national referral hospital [22]. A point prevalence study in seven countries in the Middle East performed by Alothman et al. showed that the most commonly used antimicrobials were cephalosporins (32.4%), penicillin (31.6%), carbapenems (18.8%), and glycopeptides (16.9%) [23].

The aforementioned studies indicated that third-generation cephalosporin was the most commonly used antibiotic for febrile illnesses in both in- and outpatient settings. Prompt definite diagnosis remains problematic, resulting in the high rate of empirical antimicrobial agent prescription

### 2.3. Challenges in Diagnosis of AUFI Aetiology

Distinguishing AUFI etiologies on presentation has been difficult, particularly when the mainstay of diagnosis is clinical history and examinations. The non-specificity of symptoms and signs and the lack of availability of diagnostic tests often lead to empirical and irrational use of antibiotics [6,7,8]. Nevertheless, some high-income countries (HICs) with advanced and rapid diagnostic investigations did not limit antibiotics prescriptions. High and inappropriate use of antibiotics was found in both HICs and low- and middle-income countries (LMICs) [24,25,26,27], mostly driven by high rates of HIV, TB and malaria among patients [20]. Rickettsiosis is the best example for this issue because of its diagnostic difficulty; thus, empirical treatment of doxycycline should be considered to prevent complications, particularly in some resource-limited hospitals [28]. Collateral damage of doxycycline, as well as cephalosporins or fluoroquinolones, in AMR is not well recognised. However, tetracyclines have been reported to cause vaginal flora suppression by an unknown mechanism and *Clostridioides difficile* diarrhea [29]. COVID-19 also needs to be included in AUFI etiologies, since respiratory symptoms may be absent in the early phase [30]. However, symptoms probably vary in different variants.

In addition, several limitations have been reported in various diagnostic tests; for example, blood culture sensitivity for *Salmonella* spp. ranged between 40% and 80%, and in dengue or rickettsiosis it is impossible to obtain both acute and convalescent sera, which results in delayed diagnosis [31]. Improved diagnosis of AUFI etiologies could help reduce inappropriate antibiotic prescriptions and improve patient outcomes [32].

The aforementioned limitation of diagnostic tests remains problematic. To help clinicians in their decision, biomarkers such as C-reactive protein (CRP) and procalcitonin play some role in distinguishing bacterial and viral infections [33]. In a study conducted in Northern Thailand, Wangrangsimakul et al. demonstrated that low CRP and white blood cell count were significant predictors of viral infection and that CRP was highly sensitive and specific for bacterial infections when comparing bacterial and viral groups. Moreover, a high procalcitonin level was sensitive in detecting bacterial infection, whereas low levels were not specific to viral infections [34]. If laboratory results are effective in ruling out a bacterial diagnosis, this can reduce antibiotic prescription and help combat the issue of AMR.

Although procalcitonin has been found to be specific to bacterial infections, it can be applied to tropical infectious diseases, such as dengue, to predict the severity. Thanachartwet et al. showed that procalcitonin and peripheral venous lactate levels were independently associated with dengue shock and/or organ failure [35]. Additionally, this study found that, when combined, they could provide a better diagnostic value for predicting shock and/or organ failure compared to the World Health Organisation (WHO) 2009 warning signs. However, a potential setback of procalcitonin is that it must be interpreted based on clinical progression, because high levels can be due to either bacterial co-infection or high severity of dengue infection.

RDTs have been shown to influence antibiotic prescription. In a study conducted on the impact of dengue RDTs on antibiotic and anti-inflammatory prescriptions in Colombia, Tello-Cajiao et al. found that antibiotics were prescribed in 3% of RDT-positive patients and 14% of RDT-negative patients, resulting in a decreased risk of prescribing antibiotics with a positive test [36]. However, the converse can also be seen in malaria RDTs, where they reduced unnecessary antimalarial use by malaria-negative RDTs. However, they still increased the untargeted antibiotic use because more antibiotics were being prescribed for patients with malaria-negative RDTs [13].

### 2.4. Antimicrobial Use in Malaria and Dengue

The treatment for malaria involves the use of antimalarials rather than antibiotics. Like antibiotics, inappropriate use of antimalarials can lead to resistance. The barriers to definite diagnosis can result in misdiagnosis and subsequently overtreatment, as displayed in Leslie et al.’s observational study in Afghanistan, where 413 of 414 patients had a negative malaria smear but 412 (99%) were prescribed an antimalarial drug [37]. The WHO 2010 guidelines implemented malaria testing at pre-treatment, which has reduced antimalarial use, but at the expense of increasing antibiotic use [32]. D’Acremont et al. conducted a study in Tanzania, which found that the introduction of RDTs reduced antimalaria prescriptions by 23% but increased antibiotic use by 23% [38]. Although the WHO guidelines were implemented, antimalarials are still administered without diagnosis confirmation, resulting in unnecessary antimalarial selective pressure [39]. Presumptive treatment is especially prevalent in high malaria transmission rates, with lack of laboratory expertise and without availability of RDTs [40].

In a different study in rural Tanzania, Njozi et al. found that predictors associated with the risk of antibiotic co-prescription with antimalarials were age groups and types of diagnosis [40]. Children aged < 5 years were more likely to be co-prescribed antibiotics, probably because they are a higher-risk group. As bacterial co-infection cannot be confidently diagnosed with bedside examination, antibiotics are currently prescribed in addition to antimalarials in children with severe malaria [41]. The type of diagnosis in this study [40] referred to RDT, whether it was positive, negative or not tested. Co-prescription was observed more frequently in patients with a negative malaria test or in those who were not tested.

Another factor to consider in malaria resistance is the implementation of over-the-counter antimalarial prescriptions in some countries, such as Kenya, which eventually result in overuse, as highlighted by its popularity even in areas with low and seasonal malaria transmission [42]. Abuya et al. suggested an impact of underdosing on the development of malarial resistance when over-the-counter drugs are prescribed.

**Figure 1:**
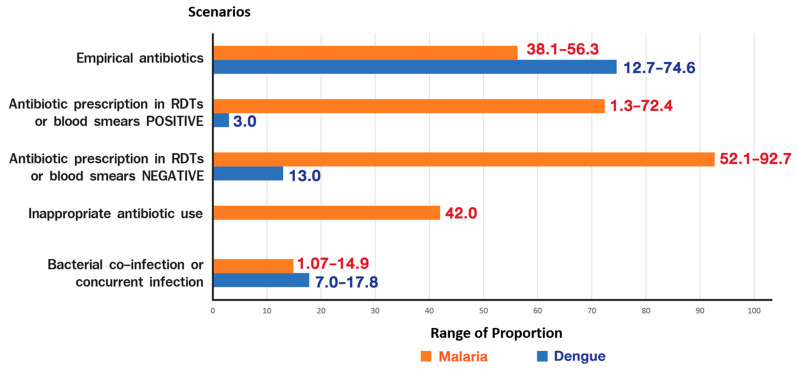
Range of scenarios related antibiotics prescription data in malaria and dengue [3,13,36,43,44,45,46,49,50,63,64,65,66,67,68,69,70] (Figure by Mrs. Siwaporn Panphoowong).

We reviewed antibiotic use in malaria and dengue, as summarised in Figure 1. We classified previous literature into the following five scenarios: the proportion of empirical antimicrobial treatment, antimicrobial prescription in positive RDTs or blood smear, antimicrobial prescription in negative RDTs or blood smear, the proportion of inappropriate antimicrobial use, and the proportion of bacterial co- or concurrent infection. This review demonstrated that the proportion of antibiotic prescriptions in dengue and malaria greatly varies throughout the literature, especially in dengue. In addition, when malaria RDTs or blood smears were negative, the proportion was higher than when they were positive.

### 2.5. Bacterial Co-Infection in Tropical Infectious Diseases

Antibiotic use in dengue or malaria would be appropriate for bacterial co-infection. The incidence of bacterial co-infection in dengue appears to be low, although the exact numbers have not been extensively elucidated. Studies report the incidence to be as low as 7% and as high as 25% [43,44]. Concurrent infections among dengue cases in Bangkok were found to be 7.8%; nevertheless, bacterial co-infection requiring antibiotic therapy was only 5.3% [3]. In a study conducted by Sunil et al. in North India, among the 124 pediatric patients with dengue fever, 10.4% had concurrent bacterial sepsis [45]. Adrizain et al. conducted a retrospective study in Indonesia and found that 17.8% of patients who tested positive for the dengue virus received antibiotics for presumed concurrent upper respiratory tract infection, typhoid fever or urinary tract infection [46]. After diagnostic testing for these presumed concurrent bacterial infections, they concluded that both the indication and choice of antibiotic in hospitalised patients with dengue were inappropriate in most patients. Kay C et al. constructed a diagnostic model (Dengue Dual Infection Score (DDISI)) for bacterial co-infection in dengue patients. The DDIS was created by five parameters for bacterial coinfection: pulse rate ≥ 90 beats/minute, total white cell count ≥ 6 × 10^9^/L, hematocrit < 40%, serum sodium < 135 mmol/L, and serum urea ≥ 5 mmol/L. The DDIS showed the satisfied validation set area and good specificity to differentiate dengue patients for empirical antibiotics [47].

Bacterial co-infection in malaria can result in higher morbidity and mortality and most commonly occur in falciparum malaria. Although both antibiotics and antimalarials are recommended in children with severe malaria, the aforementioned guidelines suggest that adults with severe malaria should not receive empirical antibiotics [41]. Bacterial and malarial co-infection rates varied among the literature in Figure 1, from 1.07% in a study from Vietnam by Phu et al. [48] to 14.9% in a study from Myanmar by Aung et al. [49] Antibiotic overuse in malaria-endemic countries should be cautiously considered, as Nyein et al. showed that nearly half of the bacterial isolates in malaria cases were resistant to empirical antibiotic treatment [50].

To our knowledge, there is no definite study on the causal relationship between inappropriate use of antibiotic and subsequently multidrug resistant (MDR) bacteria emergence in dengue or malaria. Adrizain et al. reviewed that the consequences of broad-spectrum antibiotics were the same as non-dengue patients [46].

## 3. Antimicrobial Stewardship

### 3.1. Overview of AMS

Although RDTs were generally used for the early diagnosis of dengue and malaria, several limitations have been recognised. For example, the capability of malarial RDTs for parasite detection and the intervention of temperature and humidity to the test accuracy had been observed [51,52]. Dengue RDTs provided high sensitivity and specificity, whereas the cross-reactivity and past infection detection are still controversial [53]. AMS is an additive strategy to enhance appropriate antimicrobial use in tropical diseases.

AMS refers to a coordinated programme that promotes the appropriate use of antimicrobials to improve patient outcomes, reduce AMR and decrease the spread of infections caused by multidrug-resistant organisms [54]. This term has many current definitions, but they broadly employ a coherent set of actions that promote the responsible use of antimicrobials [55]. An example of activities to describe AMS is the selection of the most appropriate antibiotic, duration, dose and route of administration for a patient with confirmed or suspected infection [56].

AMS can vary in LMICs and HICs. Initially, most research and evidence of AMS was restricted mostly to HICs but has now been redefined on a global scale [57]. LMICs, where high rates of antibiotic resistance are reported, are facing several challenges and barriers [58]. Limited resources create diagnostic challenges where correct identification of pathogens and susceptibility testing are not performed before initiating antibiotic therapy. LMICs may also face the challenge of limited access to quality-assured antibiotics or, conversely, the widespread use of non-prescribed over-the-counter antibiotics. In contrast, a study by Ashiru-Oredope et al. in England demonstrated the rapid implementation of AMS programmes in both primary and secondary care settings. They experienced high response rates from trusts across all geographical regions [59], whereas Kpokiri et al. found that strategies to combat AMR in low-resource settings are yet to be successfully implemented [60].

AMS programmes require support and collaboration from all healthcare workers. A knowledge and perception study by Adegbite et al. in Gabon found that 64% of prescribers reported awareness that AMR is an issue in their country; however, only 30% considered it an issue in their health facilities [61]. This could be explained by the lack of regular training on antibiotic prescription, which in turn can lead to overuse and eventually resistance. Rational antibiotic prescription can be achieved by altering the prescriber’s attitude and perception.

### 3.2. AMS in Tropical Infectious Diseases

Figure 1 shows the wide range of antibiotics prescribed in dengue, but these were mostly prescribed for malaria. The proportion of antibiotics prescribed among patients with malaria was higher than that with dengue. When we control the disease burden, such as the implementation of malaria-preventive measures, the incidence and antibiotic consumption declined. In a study conducted by Krezanoski et al. in malaria-endemic Uganda, extensive malaria control by means of insecticidal nets and use of indoor insecticides almost completely reduced malaria cases. As a result, a significant reduction in antibiotic treatment was achieved by 70% in 5 years post-intervention [62]. This finding is beneficial at the health system level, as it results in lower patient numbers, staffing needs and cost. The positive outcome of disease control or elimination could help reduce the spread of AMR.

RDTs affected the antibiotic consumption burden, as our review showed that the highest proportion of antibiotic prescription was observed among RDT-negative cases. Unfortunately, antibiotic consumption remained high in RDT-positive cases, emphasising that AMS implementation is urgently needed in tropical diseases. Although empirical antibiotic treatment in early AUFI cases is mandatory to reduce the mortality rate, we encourage its immediate termination whenever RDTs show positive results without evidence of bacterial infection. Bacterial co-infections or concurrent infections vary among previous literature because of different epidemiological data at the country or hospital level. Accordingly, doctors in charge should decide based on their clinical judgement, microbiological results and local epidemiological data regarding antibiotic initiation and cessation.

The evaluation of antimicrobial appropriateness of tropical diseases in previous literature is limited. Forty-two per cent of patients with malaria treated with antibiotics had no evidence of bacterial infection, whereas >10% of patients with malaria do not receive them [63]. Therefore, we strongly encourage appropriate evaluation among confirmed malaria or dengue cases in the past and present. In addition, a multidisciplinary team is crucial in AMS. This team not only evaluates and regularly monitors the antibiotic appropriateness but also implements strategies for tropical disease control. Moreover, feedback and knowledge sharing must be provided by the team to all hospital staff and stakeholders. Eventually, effective AMS should be promoted in tropical diseases with the guidance of the productive multidisciplinary team. These strategies will lead to appropriate antimicrobial use, decreased emergence of drug-resistant bacteria and elimination of tropical disease occurrence. The main ideas of AMS were summarized in the aspects of diagnosis and treatment of malaria and dengue in Table 2.

## 4. Conclusions

Previous studies showed a wide range of antibiotic use in tropical diseases, particularly in dengue and malaria. RDTs are the supporting tools for the definite diagnosis in early AUFI to limit the overuse of empirical antibiotics. However, RDTs have several limitations; thus, an AMS programme with a multidisciplinary team is another strategy to control antimicrobial inappropriateness. Clinical judgement, microbiological evidence and local epidemiological data are the factors used to determine appropriate empirical treatment. Rapid antibiotic cessation, including disease control or elimination, is the mainstay of AMS in tropical diseases. We encourage local and international sectors to foster regular antimicrobial use evaluation in tropical diseases since an AMS program is useful to AMR reduction.

## Figures and Tables

**Table 1:** Common differential diagnosis in acute undifferentiated febrile illness (AUFI).

Diseases	Laboratory Diagnosis (CDC)	Rapid Diagnostic Tests (RDTs)		Specific Treatment
		Sensitivity	Specificity	
Dengue [14,15,16]	Nucleic acid amplification tests (NAATs) Serological tests	26.9–96.7	58.3–100	None
Malaria [17]	Microscopic examination	98.37–99.10	45.47–93.33	Antimalarial agents
Influenza [18]	Reverse transcription polymerase chain reaction (RT-PCR) Immunofluorescence assays Viral culture	10–80	85–100	Antiviral agents

**Table 2:** Summary of antimicrobial stewardship in malaria and dengue.

Diagnosis
- Dengue and malaria are common tropical infectious diseases requiring treatment without antibiotics - Rapid and accurate diagnosis leads to limiting unnecessary empirical antibiotics. - RDTs might be useful for antibiotics restriction but there are some limitations.
**Treatment**
- Bacterial co-infection or concurrent infection vary among epidemiological data but usually range between 1.07–17.8%. - Inappropriate antibiotics use in malaria almost reached 45% - We encourage doctors to give empirical antibiotics when bacterial infection is doubtful due to mortality reduction. However, they should terminate prescriptions once the results show no bacterial infection.

## Data Availability

Not applicable.

## Abbreviations

AMR	Antimicrobial resistance
AMS	Antimicrobial stewardship
AUFI	Acute undifferentiated febrile illness
CRP	C-reactive protein
HIC	High-income countries
HIV	Human immunodeficiency virus
LMIC	Low- and middle-income countries
MDR	Multidrug resistance
RDTs	Rapid diagnostic tests
WHO	World Health Organisation
